# Nodular Human Lagochilascariasis Lesion in Hunter, Brazil 

**DOI:** 10.3201/eid2512.190737

**Published:** 2019-12

**Authors:** Flavio Queiroz-Telles, Gabriel L.O. Salvador

**Affiliations:** Federal University of Paraná, Curitiba, Brazil

**Keywords:** lagochilascariasis, neglected diseases, parasitology, parasites, helminthic disease, *Lagochilascaris minor*, Brazil, zoonoses

## Abstract

Lagochilascariasis is a rare helminthic infection caused by *Lagochilascaris minor* nematodes and found in Latin America; most cases are reported in the Amazon region. We report on a case observed in a hunter in southern Brazil and describe scanning electron microscopy results for *L. minor* adult forms.

Lagochilascariasis is a rare tropical helminthic anthropozoonotic disease caused by the nematode *Lagochilascaris minor* (*1*,*2*). Cases were described by Leiper on the island of Trinidad in 1909; since that report, several cases have been reported in tropical and subtropical zones of a few countries in Latin America, affecting mostly rural inhabitants from Mexico to Argentina, in both genders. Patients range from 2 to 67 years of age but are predominantly children and teenagers (*2*). Although the genus *Lagochilascaris* covers 6 species, *L. major*, *L. buckleyi*, *L. turgida*, *L. sprenti*, *L. multipapillatum*, and *L. minor*, only *L. minor* is related to human disease (*3*–*5*). Wild felines (*Felis onca, F. nebulosi*, and *F. pardalis*) are suspected to be the parasite’s natural reservoirs (*4*).

A 54-year-old male rural worker from the state of Mato Grosso, Brazil, on the border of the Amazon forest, sought medical attention for a 6-month history of a nodular lesion on the right side of the neck. He was a hunter and reported sporadic ingestion of domestic and wild feline raw meat, including meat from jaguars (*Panthera onca*). He was in good general health, except for a tumoral lesion measuring 10 cm in diameter surrounded by an irregular and erythematous skin surface in the left submandibular region, with fistulous tracts expelling 5–15-mm worms (Figure, panel A). We performed a skin biopsy for histopathologic studies and scanning electron microscopy (SEM) of the worms collected. 

**Figure:**
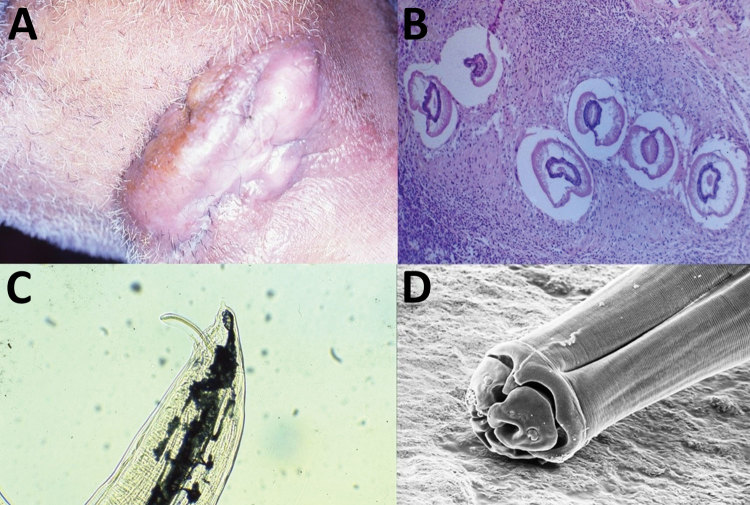
Lagochilascariasis in a 54-year-old male hunter in Brazil. A) Nodular tumorous lesion on cervical region caused by *L. minor* infection. B) Biopsy specimen of nodular lesion showing granuloma and larvae of *L. minor* nematode. Hematoxylin and eosin staining. Original magnification ×50. C) Optic microscopy shows the ejaculatory duct with spicule and lateral alae of *L. minor*. The ratio of ejaculatory duct length to spicule length of the worms is ≈2. Original magnification ×50. D) Scanning electron microscopy of the anterior end of *L. minor* nematode showing 2 subventral lips, 1 subdorsal lip, 2 interlabia, postlabial groove, papillae, and 1 amphidial pore. Original magnification ×800.

We tentatively identified the worms as *L. minor* nematodes on the basis of the following features. The skin biopsy showed multiple sinus tracts containing eggs measuring 50–90 μm and having thick shells with coarse, pitted surfaces. Larval fragments were also observed in a granulomatous reaction (Figure, panel B). Optic microscopy of 1 adult worm showed the ejaculatory duct in the posterior surrounded by spicules; the ratio of the ejaculatory duct to the spicule length was ≈2, strongly suggestive of *L. minor* (Figure, panel C).

SEM of the anterior end of the worms showed 2 subventral lips, 1 subdorsal lip, 2 interlabia, 1 postlabial groove, papillae, and 1 amphidial pore (Figure, panel D), compatible with previous reports of *L. minor* nematodes (*1**–**5*). The 3 lips were concentrically located around the oral opening.

Eosinophil count in the peripheral blood, together with biochemical and hematologic laboratory examinations, showed no abnormalities. Results of imaging evaluation of the patient’s chest and skull were also normal. We treated the patient with levamizole (300 mg/d); after 1 week, improvement in the inflammatory signs and a reduction of the purulent discharge were seen. We performed surgical resection of the lesion and continued administering levamizole at the same dosage for 2 more weeks. We then decreased the dosage by half for another 2 weeks. Follow-up biopsies showed improvement of the inflammation and absence of worms and eggs.

More than 100 human cases of lagochilascariasis have been reported (*5*). Most cases were characterized by cervical, mastoid, middle ear, pharynx, and brain nodules (*5*–*7*).

Recently, several studies have proposed that when wild cats (definitive hosts) ingest infecting eggs orally, the parasites do not reach sexual maturity (*7*). Other studies have proposed that when felines ingest rodent carcasses infected with third-stage (L3) larvae, larval hatching from cysts occurs in the stomach (*5*). After hatching, larvae migrate to upper regions of the digestive tract, reaching the adult stage in tissues of the nose and oropharynx. Some studies also suggested the idea of autoinfection, as many biopsy studies found larvae in several stages of development and eggs (*8*,*9*). The uncommon eating habits of this patient corroborate the theory of infection resulting from ingestion of raw feline meat with L3 larvae together with an autoinfection process.

SEM of *L. minor* nematode, as described in a study by Lanfredi et al. (*10*), shows the anterior end with 2 subventral papillae and lips with 1 dorsal papilla, 1 amphidial pore, and triangular interlabial prolongations (*10*). The longitudinal ventral view of the anterior region shows an excretory pore and a lateral line. The lateral view of the lips shows a deep groove around the lips forming the interlabial projection, 1 subventral lip with 1 papilla, and 1 amphidial pore (*10**,**11*). Morphologic features suggestive of *L. minor* are provided (Appendix Table).

Treatment for lagochilascariasis involves thiabendazole, cambendazole, mebendazole, albendazole, praziquantel, and invermectine (*8*,*9*). Most series report initial treatment with thiabendazole, followed by diethylcarbamazine or mebendazole and, finally, levamisole (*9*). Most reports describe recurrent and refractory infections, often because when the presence of *L. minor* nematodes is reduced and the lesion heals, physicians consider the infection resolved (*8*). However, relapses occur when inadequate treatment is given, because of the autoinfective life cycle. Although the life cycle of *L. minor* nematodes is still unknown, patients should be treated for >1 month after the clinical cure to avoid relapses.

Appendix Morphologic features suggestive of *Lagochilascaris minor* nematodes.
