# Supplementary material for: Nodular Human Lagochilascariasis Lesion in Hunter, Brazil
Source: Emerg Infect Dis. 2019 Dec;25(12):2331–2. doi: 10.3201/eid2512.190737 (PMC6874255; doi:10.3201/eid2512.190737)
Supplement: Appendix — Morphologic features suggestive of Lagochilascaris minor nematodes. [file 19-0737-Techapp-s1.pdf]

Article DOI: https://doi.org/10.3201/eid2512.190737

Nodular Human Lagochilascariasis Lesion in Hunter, Brazil

Appendix

Appendix Table. Morphological features useful for the diagnosis of *Lagochilascaris minor.*

Feature	Description
Length (mm) (*1*)	9 (male); 15 (female)
Ratio of ejaculatory duct length/spicule length (*1*)	>1
Morphology of the eggs (*1*)	Thick shells with 15–25 coarse pits
SEM morphology of the anterior end (*2*)	Dorsal lip harboring 2 subdorsal papillae and subventral lips with 1 ventrolateral papilla, 1 small papilla, and 1 amphidial pore, triangular interlabial prolongations. Excretory pores may be seen in lateral views.
Ancillary features (*1*)	• Lateral alae supported by cuticular bars extending from the nerve ring region for the whole length of the body.<br>• Striated esophagus throughout its length and gradually widened toward the posterior end.<br>• Lateral phasmids between the subdorsal and subventral papillae.<br>• Males with smooth and rounded tips. Postcloacal papillae somewhat variable in number and distribution.<br>• Females with a pair of prominent phasmids on each side in a laterodorsal position. Vulva usually behind the middle of the body.

References

1. Sprent JFA. Speciation and development in the genus *Lagochilascaris.* Parasitology. 1971;62:71–112. https://doi.org/10.1017/S0031182000071316

2. Campos DMB, Barbosa AP, Oliveira JA, Tavares GG, Cravo PVL, Ostermayer AL. Human lagochilascariasis—a rare helminthic disease. PLoS Negl Trop Dis. 2017;11:e0005510. https://doi.org/10.1371/journal.pntd.0005510
